# Endoscopic Transorbital Approach to Skull Base Lesions: Implications, Limitations, and Future Perspectives

**DOI:** 10.3390/cancers18213443

**Published:** 2026-10-24

**Authors:** Clayton Rawson, Gillian Ofori-Ntiamoah, Michael Karsy, Mehrdad Pahlevani

**Affiliations:** 1 Department of Cell Biology and Physiology, Brigham Young University, Provo, UT 84602, USA; 2 School of Medicine, Western Michigan University, Kalamazoo, MI 49007, USA; 3 Department of Neurosurgery, University of Michigan, Michigan Medicine, Ann Arbor, MI 48109, USA; 4 Department of Neurosciences Critical Care Medicine, AdventHealth, Orlando, FL 32803, USA

**Keywords:** endoscopic transorbital approach, spheno-orbital meningioma, skull base surgery, minimally invasive neurosurgery, cranial base anatomy

## Abstract

Background/Objectives: The endoscopic transorbital approach (ETOA) has emerged over little more than a decade as a minimally invasive corridor to the anterior and middle cranial fossae, reaching territory lateral to the optic nerve and internal carotid artery that is difficult to access endonasally. Its flagship indication is the spheno-orbital meningioma, where the corridor aligns with the pathology and functions best as a planned, often decompressive strategy rather than a route to radical resection. This narrative review synthesizes the current evidence and defines the approach’s role, limitations, and priorities. Methods: PubMed/MEDLINE, Scopus, and Web of Science were searched for English-language anatomical studies, clinical series, systematic reviews, and meta-analyses on the transorbital approach published between January 2016 and August 2026. Results: The evidence remains dominated by small, retrospective, single-center series and a handful of recent meta-analyses. Reported short-term outcomes—favorable proptosis relief, acceptable visual and cranial-nerve results, low pooled complication rates, and short recovery—are encouraging but constrained by short follow-up, heterogeneous nomenclature, and outcome reporting that grades residual tumor globally rather than by anatomical compartment. Gross total resection rates are lower than after transcranial surgery, and oncological equivalence has not been established. Technical, reconstructive, ophthalmic, and anatomical factors define the corridor’s ceiling, and ETOA is best positioned against endonasal and transcranial alternatives as a complementary rather than competing tool. Conclusions: ETOA is an established corridor whose maturation depends on reconstructive refinement, a flatter learning curve, standardized terminology and outcome sets, and prospective comparative data—consolidation rather than anatomical expansion.

## Simple Summary

Tumors that grow where the eye socket meets the skull base, most often a type called spheno-orbital meningioma, can push the eye forward and threaten vision. Removing them through a traditional open operation requires a large incision, detachment of muscle, and some retraction of the brain. The endoscopic transorbital approach reaches these tumors through a small incision in the eyelid or around the eye, avoiding a formal opening of the skull. This review summarizes what is currently known about the technique: which tumors it suits best, how safe it is, and where its limits lie. In the short term, the approach relieves eye bulging well and preserves vision in most patients, but it usually removes less tumor than open surgery, so it is best used as a planned, function-preserving operation. Larger, longer, and better-standardized studies are needed to define its role.

## 1. Introduction

Over the past three decades, skull base surgery has been reshaped by the recognition that operative morbidity depends as much on the corridor chosen to reach a lesion as on the lesion itself [1]. Conventional transcranial routes to the anterior and middle cranial fossae provide generous exposure, but at the cost of soft-tissue dissection, temporalis mobilization, variable brain retraction and trans-cisternal dissections, and a cosmetic penalty for the patient. In spheno-orbital meningioma (SOM), for example, pooled microsurgical transcranial data from 1903 patients across 59 retrospective studies yield gross total resection (GTR) in 59.8% of cases (95% CI 49.5–70.2) alongside focal cranial nerve deficit in 21.0% (95% CI 14.5–27.6) [2], and the same exposure-versus-morbidity trade-off recurs in multicenter experience with extensive tumors [3].

Endoscopic endonasal surgery addresses one part of this problem, offering a direct route to ventral midline and paramedian disease [1,4]. Its limitation, however, is anatomical rather than technical: as pathology extends laterally, beyond the optic nerve and paraclinoid internal carotid artery into the lateral cavernous compartment, middle fossa, Meckel’s cave, lateral sphenoid wing, petrous apex, and temporal region, the endonasal surgeon must work across the structures that require protection rather than along them [4,5,6]. Cadaveric work confirms that endonasal access to the lateral cavernous sinus is severely constrained by the tortuous intracavernous course of the carotid artery [7].

The ETOA was designed for precisely this anatomy. Entering through one of the four orbital quadrants and removing a selected portion of the orbital wall, it creates a short trajectory to the anterolateral skull base along the axis of the middle fossa floor, sparing craniotomy and temporalis mobilization and requiring minimal brain retraction [5,8]. It is best understood not as a competitor to the endonasal route but as its lateral counterpart: the two corridors meet at defined connection zones and can be deliberately combined [9,10].

The corridor’s principal trade-off is the extent of resection. In the only meta-analysis directly comparing transorbital and transcranial surgery for SOM (53 studies, 2016 patients, of whom 155 [7.7%] underwent ETOA), the pooled GTR rate was 21.1% for ETOA versus 48.0% for transcranial surgery, with subtotal resection correspondingly more frequent after ETOA [11]. Because the pooled cohorts were retrospective, non-randomized, and unadjusted for tumor extent, this difference reflects case selection as well as approach and should not be read as a causal effect of the corridor. The direction of this effect is consistent across analyses, even where its magnitude cannot be estimated precisely. Whether the additional residual carries an oncological price is at present unresolved: reported ETOA follow-up is short (median 18 months) relative to transcranial series (median 52 months), and pooled progression-free survival for surgically treated SOM declines from 75.5% at five years to 49.1% at ten [2,11]. The low early recurrence rates reported after ETOA are therefore not yet interpretable as measures of durable tumor control. As Section 6 and Section 7 develop, the appropriate response to this uncertainty is neither to withhold the corridor nor to over-read favorable short-term data, but to adopt it explicitly as a planned, decompression-oriented strategy in anatomically selected tumors, with the adjuvant plan defined before rather than after surgery.

## 2. Materials and Methods

A narrative rather than a systematic format was chosen deliberately. Five systematic reviews and meta-analyses of the transorbital approach have been published since 2023 [2,11,12,13,14], and the primary series they pool overlap substantially; a further quantitative synthesis of the same retrospective, single-arm data would add little precision while inheriting the same heterogeneity in nomenclature, outcome definitions, and follow-up. The aims of this review are instead interpretive and integrative: to set the existing syntheses side by side and reconcile their estimates (Table 1); to compare the transorbital, endonasal, and transcranial corridors across anatomical, technical, and evidentiary attributes (Table 2); to translate the evidence into a practical framework of indications and contraindications (Section 6.9, Table 3); and to propose a compartment-specific standard for reporting residual tumor, ophthalmological outcomes, and long-term tumor control (Table 4), which the existing quantitative syntheses identify as missing but do not supply. The manuscript was structured with reference to the SANRA scale for the quality of narrative review articles [15].

This is a narrative review and did not employ a formal quality or risk-of-bias assessment; its findings should be read as descriptive. PubMed/MEDLINE, Scopus, and Web of Science were searched for English-language articles published between January 2016 and August 2026, combining the terms “transorbital,” “endoscopic transorbital approach,” “transorbital neuroendoscopic surgery,” “superior eyelid,” “lateral orbitotomy,” and “skull base,” supplemented by hand-searching of reference lists from retrieved reviews, meta-analyses, and clinical series. Landmark publications predating 2016 are discussed where historically necessary but are cited through subsequent syntheses. Anatomical studies, clinical series, systematic reviews, and meta-analyses were eligible; single case reports and pure microscopic transorbital approaches were used only to document indications not otherwise represented.

Study selection proceeded in two stages: screening of titles and abstracts for relevance to endoscopic or endoscope-assisted transorbital access to the skull base, followed by full-text review of potentially eligible articles. Articles were included if they were peer-reviewed and reported (i) cadaveric, morphometric, or radiological anatomical data on transorbital corridors; (ii) clinical outcomes of endoscopic transorbital surgery for skull base or intracranial lesions; or (iii) a systematic review or meta-analysis of such outcomes. Articles were excluded if they were conference abstracts, editorials, or letters without original data; described purely intraorbital surgery without skull base or intracranial extension; described penetrating transorbital injury; or were not available in English. Where several publications reported overlapping cohorts from the same institution, the most recent or most complete report was preferred. Pooled estimates were taken from the published meta-analyses, with their denominators and confidence intervals where available, rather than recalculated from primary series, and no new pooling was performed. Because the included clinical literature is predominantly retrospective, single-arm, and non-randomized, differences between approaches are presented as reported and interpreted in light of case mix rather than as causal effects.

## 3. Evolution of the Technique and the Terminology Problem

The modern transorbital corridor grew out of transorbital neuroendoscopic surgery (TONES), formally proposed in 2010 as a family of endoscopic routes crossing the four orbital quadrants to reach the anterior and middle skull base [25,29]. Its conceptual novelty was to reframe the orbit from a structure to be preserved during skull base surgery into the route through which that surgery is performed.

Between 2016 and 2018, a series of anatomical studies established feasibility from a neurosurgical standpoint: interdural dissection from the meningo-orbital interface exposed the lateral cavernous sinus [30]; the superior eyelid approach was characterized in detail [31]; the lateral corridor to Meckel’s cave was validated [32]; the petrous apex was shown to be reachable transorbitally [33]; and endo- versus extra-orbital corridors to the spheno-orbital region were compared [34]. Clinical experience followed rapidly in selected SOM [35], cranio-orbital tumors [36], hyperostotic SOM [16], and Meckel’s cave and middle fossa tumors [37]. Within a further five years the reported scope had widened to optic canal decompression [18], sphenoid wing meningiomas invading the cavernous sinus [38], the cavernous sinus itself [7], the insular and mesial temporal regions [39], the middle incisural space [40], petroclival lesions with or without anterior petrosectomy [20], and pediatric skull base pathology [41]. By the mid-2020s the approach had generated its own systematic reviews and meta-analyses [5,12,13,14,29].

Despite this rapid diffusion, terminology remains inconsistent. The same operation is variously called TONES, the ETOA, the superior eyelid endoscopic transorbital approach (SETOA), the lateral transorbital approach (LTOA), and the lateral orbital wall approach, with these labels used interchangeably as synonyms, as subsets of one another, and as distinct operations [5,8]. The most stable scheme is the four-corridor concept, in which the approach is named for the orbital quadrant of entry [5,8]; a second, less consistent scheme grades the degree of expansion of the standard corridor, with “extended,” “open-door,” and “supero-lateral orbital rim osteotomy” applied variably [8,42].

Three standardization efforts merit attention. Di Somma and colleagues proposed five ascending levels of difficulty, from extraconal and extradural work up through intradural and intraconal dissection, the interdural approach to Meckel’s cave, the opticocarotid–cavernous region, and finally the petrous apex, posterior fossa, insula, and Sylvian fissure [21]. Mathios and colleagues classified the lateral transorbital approach by seven anatomical target zones: the orbit; the lesser sphenoid wing and anterior clinoid; the middle fossa; the lateral cavernous wall and Meckel’s cave; the infratemporal fossa; the petrous apex; and the anterior fossa, and related complications and ocular outcomes to the operative target [26]. De Notaris and colleagues distinguished “standard” from “extended” approaches, subdividing the latter into extensions that widen the proximal corridor (improving maneuverability) and those that widen the distal corridor (granting access to deeper compartments), which may be combined according to target [25]. An independent group applied the difficulty scale in practice, classifying cavernous sinus work as a stage-4 procedure among the most technically demanding [7].

## 4. Surgical Anatomy, the Corridor Concept, and Its Oncological Consequences

The central conceptual advance of transorbital surgery is to treat the orbit not as a monolithic space but as four distinct corridors. The corridor of entry determines the accessible territory, and therefore the residual left by any given operation—more decisively than any subsequent step [5,8].

The superior corridor, entered through the supratarsal fold in the superior eyelid crease approach, is the most versatile for neurosurgery and accounts for most intracranial transorbital operations to date; it reaches the superior orbit, frontal sinus, supraorbital space, posterior anterior cranial fossa and, via the greater sphenoid wing, the lateral middle fossa [5,25]. The medial (precaruncular) corridor accesses the orbital roof, lamina papyracea, ethmoidal arteries, optic nerve, the parasellar and paraclinoid carotid, and the medial anterior skull base [5,8]. The lateral (retrocanthal) corridor, entered through the conjunctiva behind the lateral canthus, opens the deep lateral orbit and, after removal of the greater sphenoid wing between the superior and inferior orbital fissures, the middle fossa and the temporal and infratemporal fossae, with the optic nerve separated from the trajectory by the contents of the superior orbital fissure and the optic strut [5,8]. The inferior (preseptal lower-eyelid) corridor reaches the inferior orbit, orbital floor, maxillary sinus, inferior orbital fissure, and foramen rotundum, and is used mainly in combination with the lateral and/or medial corridors [5,8].

Navigation within the corridor relies on a consistent set of references: the lateral orbital wall as a starting landmark; the sphenofrontal suture, the sphenoid ridge with its greater and lesser wings, and the superior and inferior orbital fissures as the structural framework; and the meningo-orbital band as the partition between orbit and intracranial compartment. Codes and colleagues argue that bone provides the most reliable landmark system here, identifying five bony pillars that govern access, including the lesser sphenoid wing, the sagittal crest (the medial aspect of the greater wing), the anterior clinoid, and the middle fossa floor [43]. Sharp division of the meningo-orbital band opens the interdural space between the meningeal and periosteal dural layers, exposing the lateral cavernous wall without entering the sinus [30]; systematic anatomical study has confirmed the reliability of these techniques for the anterior and middle fossae [44].

The accessible territory spans the anterior cranial fossa, including the olfactory groove and planum [45], the spheno-orbital region and lesser sphenoid wing [34], the anterior clinoid and optic canal [46], the middle fossa floor and lateral cavernous wall [7,30], Meckel’s cave [32], the temporal pole and mesial temporal structures [39], the middle incisural space [40], the Sylvian fissure and proximal middle cerebral artery in cadaveric work [47], the infratemporal fossa, and through anterior petrosectomy, the petrous apex and, selectively, the posterior fossa [20,26,33]. Quantitative data show that removing the lateral orbital rim progressively increases the angle of attack and surgical freedom in proportion to the extent of orbitotomy [48], a relationship anticipated by earlier comparisons of rim preservation versus osteotomy for cavernous access [49] and consistent with the rationale for modular extended approaches, in which proximal extensions enhance maneuverability and distal ones enhance depth [25,42].

Where the corridor stops is clinically decisive, because these boundaries define the residual. Four anatomical constraints set the ceiling. Globe displacement is limited, conventionally to about one centimeter, capping the width of the working channel regardless of bone removal [8]. The corridor is inherently paramedian and ipsilateral, limiting access to midline and contralateral structures [8]. Work beyond the trigeminal porus is constrained by the subtemporal ridge and the slope of the middle fossa floor, so posterior fossa access is selective and usually depends on petrosectomy or a combined corridor [8,20]. Finally, the single external entry point, versus two nostrils endonasally, restricts the simultaneous use of multiple or bulky instruments and, with them, the control of significant bleeding [7,8]. A cavernous-sinus-specific caution is that the cavernous carotid lies largely concealed behind the ophthalmic division of the trigeminal nerve, so it is not identified early in transorbital work as it is endonasally [7].

Tumors involving the superior orbital fissure, cavernous sinus, medial optic canal, extraocular muscles, or cranial nerves therefore lie at or beyond the corridor’s safe margin. Crucially, however, resection in these regions is limited regardless of approach: the transcranial literature likewise converges on restraint at the superior orbital fissure and cavernous sinus because clearing them carries high cranial-nerve morbidity [2]. The transorbital corridor does not create compartments that cannot be cleared; it enlarges the volume of residual located outside them. Whether that enlargement is oncologically meaningful remains an open question that the current literature has not been designed to answer, since no dataset stratifies residual by anatomical compartment.

Stratification by tumor anatomy is nonetheless suggestive. When SOMs are grouped by class (I, lateral or superolateral; II, medial or inferomedial; III, orbital apex; IV, diffuse), class I tumors achieve GTR in 78.6% (95% CI 60.1–97.1) and visual improvement in 71.5% (95% CI 63.7–79.4), both significantly better than the pooled population, with a recurrence rate of 15.1% [2]. Class I corresponds precisely to the tumors the transorbital corridor best reaches. Because this stratification was based on tumor anatomy rather than approach, and no analysis has stratified by both simultaneously, it does not establish resection equivalence for class I disease; but it does indicate that the resection deficit associated with ETOA is not uniform across anatomical classes, and that pooled figures may understate its performance in the class for which the corridor was designed.

## 5. Surgical Technique

### 5.1. Positioning, Exposure, and Osteotomy

The patient is positioned supine with the head fixed and rotated to the contralateral side, allowing sufficient neck extension to align the surgeon’s axis with the middle fossa floor. A superior eyelid crease incision in the supratarsal fold, extended laterally as the target dictates, provides the standard access, while the retrocanthal and precaruncular routes use conjunctival incisions that leave no skin scar [5,8]. Dissection proceeds beneath the orbicularis to the orbital rim, preserving the periorbita to prevent fat prolapse into the corridor, followed by subperiosteal elevation from the roof or lateral wall according to the corridor selected; the lateral route additionally requires detachment of the temporalis from the lateral orbital wall [5,7,8]. Orientation is guided posterolaterally by the superior orbital fissure and medially by the ethmoidal bundles at the frontoethmoidal suture, which mark proximity to the optic nerve [5,8]. The cornea is protected and the periorbita retracted medially with a malleable retractor [7].

Bone removal is tailored rather than standardized: the pillar relevant to the target is taken down, most often the greater and lesser sphenoid wings for middle fossa and cavernous approaches, and the lesser wing with the optic strut for clinoidal and optic canal work [7,18,43]. Removal of the lateral orbital rim is performed selectively or routinely to improve maneuverability; it is not mandatory but reduces orbital retraction and eases the procedure [7,14,48,49]. The meningo-orbital band is then identified and sharply divided, after which the dura is elevated extradurally or opened according to pathology, with anteroposterior interdural dissection exposing the lateral cavernous wall and Meckel’s cave without formal intradural entry [7,30,37].

### 5.2. Instrumentation and the Two-Surgeon Corridor

Standard visualization uses a rigid 4 mm 0° endoscope, with angled scopes reserved for specific maneuvers [7,8]. Most teams use a two-surgeon, four-hand technique, but ergonomics are unusually demanding: unlike the two nostrils available endonasally, the transorbital route typically offers a single external portal, so surgical freedom falls sharply once endoscope, instruments, and retractor are introduced together [7]. Two solutions have been proposed and quantified: a biportal technique adding a second working portal [50], and enlargement of the orbitotomy [48,49]. The endoscope may be held either by the second surgeon, which preserves dynamic visualization and allows depth and angle to be adjusted in coordination with the operator’s instruments and suction, or by an external holding arm, which provides a stable image and frees both hands of a single operator, at the cost of a static view and an additional rigid object within an already crowded single portal [8,22]. Dynamic retraction, in which the periorbita is intermittently displaced and released, was used in five of eleven studies in the largest safety meta-analysis, versus fixed retraction in one, with the remainder unspecified [14]. Orbital access and protection of the globe are best managed collaboratively. Participation of an ophthalmologist or oculoplastic surgeon is desirable for the orbital stage of the operation (incision, subperiosteal dissection, and handling of the periorbita) and for perioperative ophthalmological assessment, a model adopted by several of the groups that developed the technique [23,36,51]. Retraction should be limited in both magnitude and duration. Intraocular pressure rises curvilinearly with medial retraction of the orbit, gradually up to approximately 1.5 cm of displacement and steeply beyond it, and continuous intraorbital pressure measurement has been proposed as a surrogate monitor [52]. A recent appraisal of retraction-related injury accordingly recommends limiting globe displacement to approximately 10–11 mm and never beyond 15 mm, applying retraction in short cycles rather than continuously, releasing it gently to avoid decompression retinopathy, and monitoring pupillary size and reactivity throughout, mydriasis being a more practical intraoperative warning sign than a relative afferent pupillary defect [24]; cautious handling of orbital tissue and monitoring of the pupillary response are likewise emphasized by experienced neurosurgical–oculoplastic teams [23]. In practice, this translates into intermittent release of the retractor at regular intervals, approximately every 20 min being a pragmatic schedule although no interval has been formally validated, with assessment of the pupil, clinically or by quantitative pupillometry where available, at each release. Reassuringly, ETOA-related oculomotor deficits were predominantly transient and typically followed extended extradural or interdural dissection near the superior orbital fissure and cavernous sinus, a pattern the authors ascribed to traction or edema rather than direct nerve injury, an interpretation consistent with, though not proven by, the pooled data [14].

### 5.3. Reconstruction and Its Difficulties

Reconstruction is where ETOA is structurally weaker than endonasal surgery, chiefly because the local vascularized nasoseptal flap is unavailable. Closure relies instead on multilayer repair, fat, fascia, and synthetic or autologous grafts, with bony reconstruction for larger defects and watertight closure whenever dissection extends beyond the orbital rim into the sphenoid sinus, cribriform plate, planum sphenoidale, or lateral sphenoid recess [5,8]. Despite this constraint, outcomes are generally favorable. Pooled cerebrospinal fluid (CSF) leak rates were 2.5% across 240 cases in a dedicated meta-analysis [12] and 1% (95% CI 0–4%) across 174 patients in another (i.e., the eight of eleven included studies that reported this outcome, of 269 patients pooled overall; Table 1), rising to 6% in the meningioma-only subgroup (95% CI 2–14%; four studies, 92 patients) [14]. In SOM specifically, the pooled leak rate after ETOA (5.0%) did not differ significantly from transcranial surgery (4.9%) [2], and individual small series report either no leaks [22] or isolated leaks that resolved with lumbar drainage [53]. The problematic configuration is the combined transorbital–endonasal approach, with a pooled SOM leak rate of 20.3% (95% CI 0–46.7%); the confidence interval spans nearly the entire plausible range and the between-group comparison was not significant (*p* = 0.551), so this is best read as a signal warranting attention rather than an established finding [2]. Reconstruction is thus adequately solved for the single corridor but remains to be worked out for multiportal surgery.

### 5.4. Intraoperative Adjuncts

Neuro-navigation is widely used and is particularly valuable when an unfamiliar target is approached through an unfamiliar corridor. Cadaveric and clinical transorbital work has been performed under navigation registered to preoperative CT [7], and lesion-specific preoperative planning of bone openings is among the factors associated with procedural success [6,29]. Volume rendering of tumor and adjacent neurovascular structures can simulate transorbital and endonasal views before multiportal surgery, and intraoperative CT between stages has been used to estimate residual tumor volume before opening the second corridor [7]. Micro-Doppler assists in localizing the cavernous carotid, which, concealed behind the ophthalmic nerve, is not visualized early [7]. Given the corridor’s proximity to the oculomotor, abducens, and trigeminal nerves, intraoperative neuromonitoring is a logical adjunct: triggered electrooculography has been described for identifying the oculomotor and abducens nerves during skull base surgery [54], and blink-reflex monitoring for the trigeminal nerve [55].

## 6. Indications, Surgical Objectives, and Case Selection

### 6.1. Spheno-Orbital Meningioma and a Shift in Objective

SOM is the most frequent indication and the core of transorbital practice, comprising 60.6% of pooled contemporary cases [14]. This predominance follows from the anatomy: SOMs arise at the terminus of the transorbital corridor, and their characteristic proptosis and visual decline stem from hyperostosis and orbital apex compression rather than intracranial mass effect. Several independent series document the surgical experience [16,17,35,36,56].

As outlined in Section 1, the comparative data must be read with their limitations in view: the two meta-analyses draw on retrospective, non-randomized, largely single-center series without case-mix adjustment, and the transorbital cohorts are small (155 of 2016 patients in one) and skewed toward tumors with limited intradural extension [11]. Selection bias is therefore likely to be substantial and to run in a consistent direction: transorbital cohorts are enriched for laterally located, often hyperostosis-dominant tumors, whereas transcranial series span the full range of tumor extension and anatomical complexity, including multicompartmental and extensive intradural disease [2,3,11]. Neither analysis could adjust for tumor class, volume, baseline visual and proptotic status, or surgeon experience, so pooled between-approach differences, in either direction, conflate the effect of the corridor with the effect of case mix. With that proviso, three findings recur. Pooled proptosis relief was higher in the ETOA cohorts (96% vs. 72% in one analysis; 79.4% vs. 60.0% in the other) [2,11]. Baseline proptosis and hyperostosis burden were not adjusted for, however, and this difference should be regarded as hypothesis-generating rather than as evidence of superiority. Visual improvement does not differ significantly between approaches, though the divergent absolute estimates across analyses reflect differing inclusion criteria and outcome definitions and are themselves a caution about pooled functional data in this literature [2,11]. Resection completeness favors transcranial surgery, as detailed in Section 1 [2,11]; a single-center comparison with the extended mini-pterional approach and case series with systematic review concur [22,57,58,59].

Two further findings that might seem to favor ETOA do not survive testing and should not be claimed as advantages. Focal cranial-nerve deficits were 7.3% after ETOA versus 21.0% after transcranial surgery, without a significant difference (*p* = 0.411) [2], and the overall complication rate did not differ (18% vs. 26%, *p* = 0.165) [11]. The complication profiles do differ descriptively: transorbital series reported a narrower spectrum dominated by diplopia, ptosis, and trigeminal sensory disturbance; transcranial series reported a broader one including visual deterioration, vascular events, infection, and CSF leak, but this difference was not tested statistically [11]. Dedicated ophthalmological assessment provides the granular ocular data that pooled analyses lack [27].

Judged solely by GTR, ETOA looks unfavorable. Judged against the real morbidity of these tumors, its short-term results are consistent with a favorable trade-off in selected patients, although this remains to be confirmed by adjusted comparative data with long-term follow-up—and reflect a shift in objective that predates ETOA and arose within the transcranial literature itself, where several groups independently concluded that decompressive rather than Simpson-grade resection is appropriate for en-plaque and hyperostotic SOM, given the disproportionate morbidity of clearing tumor from the superior orbital fissure, cavernous sinus, and extraocular muscles [2]. ETOA does not initiate this shift; it operationalizes it.

### 6.2. Open Questions and Their Answerability

That operationalization leaves a larger residual burden after transorbital surgery, which remains to be characterized. It would be equally unjustified to assume this residual is oncologically inert or that it is not. Two considerations temper alarm: the regions where resection stops are similar for both approaches, and pooled analysis shows no significant difference in five- or ten-year progression-free survival between them [2]. Against this, those pooled estimates rest on 230 and 159 patients respectively, are not stratified by transorbital exposure, and derive from follow-up (median 18 months; mean 27.6 ± 15.1 months) far shorter than the interval over which PFS falls from 75.5% to 49.1% [2,11,14]. Until adjusted, long-term data are available, the lower GTR rate after ETOA should be treated as a real difference whose oncological consequence is unknown, and oncological equivalence with transcranial surgery should not be assumed.

The question is nonetheless answerable; the obstacle is a reporting convention, not a fundamental limit. Extent of resection is reported as a global grade, gross total, near total, subtotal, or partial, with variable definitions [14], but the compartment in which residual lies is not. If the extra residual left by the transorbital corridor is hyperostotic bone or dural tail, its natural history differs from that of residual tumor in the cavernous sinus or orbital apex. We identified no series reporting postoperative residual by anatomical compartment; until such reporting exists, cross-approach comparisons will remain underdetermined however long patients are followed. The request is tractable: pre- and postoperative volumetric imaging has already been used in a transorbital series [26], intraoperative CT has quantified residual between multiportal stages [7], and an adequate vocabulary for localizing residual already exists in the target-zone and modular classifications [25,26]. A minimum dataset that would make residual tumor, ophthalmological outcome, and long-term tumor control comparable across series is proposed in Section 10 (Table 4).

### 6.3. Adjuvant Integration

If subtotal resection is anticipated, adjuvant therapy could be planned before surgery. Current practice is neither standardized nor consistently reported: across pooled SOM data there is no uniform radiotherapy protocol, residual WHO grade 1 tumors are generally observed irrespective of Simpson grade, recurrent grade 1 tumors are usually managed by repeat surgery with postoperative radiotherapy, and adjuvant radiotherapy is regarded as mandatory for grades 2 and 3 [2]. Subtotally resected meningiomas respond well to radiotherapy, with higher overall and progression-free survival than surgery alone, and stereotactic radiosurgery, alone or with hypofractionated radiotherapy, is well suited to residual disease near the cavernous sinus and orbital apex, precisely the compartments the transorbital corridor cannot safely clear [2]. A corridor that leaves predictable residual in a predictable compartment is therefore well matched to multimodality planning, provided that planning precedes surgery. Two reporting deficits impede evaluation, however: WHO grade was recorded in only 31 of 59 studies (52%) in the largest SOM review, and adjuvant treatment was inconsistently documented [2]. Recording both should be mandatory in future series, since without them residual disease cannot be risk-stratified.

### 6.4. Recurrent and Re-Operative Cases

Recurrent SOM warrants separate consideration. Pooled recurrence across all approaches is 20.7% among 1409 patients [2], and re-operation after prior transcranial surgery is technically demanding. The transorbital corridor offers an unscarred route to the tumor: Ricciuti and colleagues reported five consecutive recurrent cases managed via a modified eyelid crease approach without postoperative CSF leak and with no new deficit beyond a pre-existing lateral rectus palsy. These establish feasibility rather than efficacy, and no comparative data exist for re-operative transorbital surgery. A cautionary observation from the multiportal literature is relevant: after previous craniotomy and repeated radiosurgery, dense fibrosis prevented clean interdural dissection, and the surgeons proceeded instead through a window in the anteromedial triangle [7]. Even so, the logic of reserving a fresh corridor for the reoperation these tumors frequently require is coherent, is strengthened by the expectation of subtotal primary resection, and deserves prospective study.

### 6.5. Other Neoplastic Indications

Trigeminal schwannoma is the second most frequent pathology in ETOA series (12.0%) [14]. The corridor reaches Meckel’s cave along an interdural plane validated anatomically before clinical use [32] and subsequently applied to Meckel’s cave and middle fossa tumors [37]; transorbital and endonasal routes have been compared for this pathology, with corridor choice governed by tumor extent [60], a comparison extended to middle fossa tumors more broadly [61] and synthesized in a dedicated systematic review [62]. Being circumscribed rather than en-plaque, trigeminal schwannomas do not share the resection deficit of SOM, and GTR is a valid objective. Beyond these, ETOA has been applied to anterior and middle fossa meningiomas including anterior clinoidal [25] and olfactory groove lesions [45], to optic canal decompression for compressive optic neuropathy [18,46], to sphenoid wing meningiomas invading the cavernous sinus [7,38], and to broad institutional series of orbital and skull base lesions [63,64,65]. At the upper end of technical difficulty, 32 petroclival tumors treated by transorbital transcavernous approaches with or without anterior petrosectomy achieved gross total or near-total resection in 28 cases over a median 34.7 months [20]. The corridor also reaches the temporal pole, mesial temporal structures, and insula [39,40], raising the prospect of application to selected temporal-pole gliomas, though the evidence there remains largely anatomical and early clinical [8]. Pediatric transorbital series have also been reported [41]. These higher-difficulty applications remain, on current evidence, the territory of high-volume centers [14,21].

### 6.6. Non-Neoplastic Applications

Although this review centers on neuro-oncology, non-neoplastic applications are substantial and have shaped the approach’s development. A systematic review finds that CSF leak and encephalocele repair, inflammatory and infectious lesions including abscess drainage, orbital roof and anterior skull base trauma, and orbital decompression together account for a considerable share of published cases [5]. Optic nerve decompression for non-neoplastic compressive optic neuropathy uses the same lateral-to-medial corridor validated for tumoral compression [46], and institutional series encompass both categories within one technical framework [65]. These carry two implications for oncological practice: the reconstructive experience gained through non-neoplastic work is the same on which tumor closure depends, and non-neoplastic cases have been the principal route by which surgeons acquire the technique before applying it to tumors.

### 6.7. Vascular Applications

Vascular applications remain occasional rather than established. The best-defined indication is carotid-cavernous fistula, where the orbital route allows venous access to the cavernous sinus when endovascular approaches fail or are unavailable, as reviewed systematically [66]. Anatomical study has demonstrated transorbital exposure of the Sylvian fissure, proximal middle cerebral artery, and crural cistern [47], and the cavernous carotid can be exposed and confirmed by Doppler during tumor surgery [7]. The governing limitation is the single external portal and consequent restriction on simultaneous instrument use, which constrains hemorrhage control [7,8]. Extension to vascular pathology therefore demands greater caution than extension to tumors, and no clinical series supports routine transorbital aneurysm surgery.

### 6.8. Combined and Multiportal Strategies

For lesions spanning medial and lateral territory, the transorbital and endonasal corridors are complementary. Quantitative anatomy has defined their zones of convergence—the sphenoid planum anteriorly, Mullan’s triangle in the middle fossa, and the region just behind the medial petrous apex posteriorly—and compared the working areas each corridor affords there [9]. Clinical experience with combined surgery, though limited in volume, reports adequate access and acceptable complication rates for paramedian tumors [10]; a representative case is the staged removal of a recurrent invasive pituitary adenoma, in which the endonasal corridor cleared the intrasellar and suprasellar compartments while the lateral cavernous compartment, unreachable endonasally because of the tortuous carotid, was addressed transorbitally through the anteromedial triangle [7]. Two cautions attach. Reconstructively, combined surgery carries the highest pooled CSF leak rate among SOM configurations (20.3%), albeit with a very wide, non-significant interval [2]. Evidentiarily, meta-analyses routinely exclude multiportal cases to isolate the transorbital corridor [14], which systematically underrepresents combined experience in the very safety data by which the approach is judged.

### 6.9. A Selection Framework

Four questions, asked in sequence, capture the empirical basis of case selection. First, what is the lesion’s relation to the optic nerve and internal carotid artery? Lateral targets favor the orbit, medial and inferomedial targets the nose, and lesions crossing that axis a combined corridor [5,9,10]. Second, within SOM, what is the anatomical class? Class I lateral and superolateral lesions offer the best resection and functional outcomes and the closest fit to the corridor [2]. Third, what is the operative objective? Decompression, diagnostic access, and functional preservation are well served; cytoreduction of bulky, multicompartmental, or extensively dural-based tumors is not, and the resection statistics confirm rather than contradict this [2,11]. Fourth, what are the tumor’s characteristics? Bulky, firm, or highly vascular lesions, and any with vascular encasement, sit poorly with a corridor that limits both instrument exchange and hemorrhage control [7,8].

Table 3 condenses these considerations into practical indications and relative contraindications, set against the transcranial alternative. The contraindications are relative rather than absolute: most reflect the corridor’s anatomical ceilings (Section 4) and its limited capacity for hemorrhage control and reconstruction (Section 8) rather than formal comparative evidence, and the comparison with transcranial surgery rests on non-randomized cohorts that differ in tumor extension and anatomical complexity.

## 7. Outcomes and the Current Evidence Base

The clinical case for ETOA rests on a body of evidence that is growing quickly but remains, by the standards of comparative surgery, immature. Almost all published experience takes the form of single-arm, retrospective, single-center series concentrated in a small number of pioneering groups, and the systematic reviews and meta-analyses that now anchor the field pool these series rather than compensate for their design (Table 1) [5,8,29]. The largest safety synthesis to date pooled eleven observational studies, two prospective and nine retrospective, published between 2019 and 2025, comprising 269 patients, and its authors concluded explicitly that prospective comparative studies are still required to refine indications and validate long-term efficacy [14]. That conclusion, rather than any single effect estimate, is the most important result the outcome literature currently supports.

In appraising this literature, three questions should be kept distinct, because the evidence for each differs in kind and maturity. Anatomical feasibility, whether a target can be reached and manipulated through the corridor, is established by cadaveric and morphometric studies and is the most mature level of evidence [7,9,30,31,32,33,34,48]. Short-term functional outcomes, namely proptosis, visual function, ocular motility, and complications in the first months to two years after surgery, are supported by retrospective clinical series and their meta-analyses and are encouraging [2,11,12,13,14]. Oncological efficacy, durable tumor control relative to the extent of resection achieved, is the least developed: GTR rates after ETOA are consistently lower than after transcranial surgery [2,11], follow-up (median 18 months) is far shorter than the interval over which meningioma progression declares itself [11], and no study has compared progression-free survival between approaches after adjustment for tumor extent. Feasibility and favorable short-term function should therefore not be read as evidence of oncological equivalence, which remains unproven.

On extent of resection, the pooled picture is internally consistent across analyses. Single-arm meta-analysis places gross total resection near the middle of the reported range, with subtotal resection common [14], and the only head-to-head SOM comparison confirms lower GTR after ETOA than after transcranial surgery, as detailed in Section 1 and Section 6 [2,11]. The interpretive weight of these figures depends entirely on the operative objective assumed: read as attempts at radical excision they are disappointing, but read as planned decompressive procedures in anatomically selected tumors, the objective most transorbital series actually adopt, they are broadly consonant with intent.

Functional and cosmetic outcomes are where the corridor shows to best advantage. Proptosis relief is consistently high and, in unadjusted comparative data, is higher after ETOA than after transcranial surgery, a difference that may partly reflect case selection [2,11]; visual improvement occurs in roughly half of patients in pooled single-arm data and does not differ significantly from transcranial results [2,11,14]. Ocular morbidity is the natural counterweight and is examined in Section 8, but dedicated ophthalmological follow-up and target-zone analyses indicate that the commonest deficits, diplopia and gaze restriction, are frequently transient and concentrate in the more extensive cavernous and superior orbital fissure dissections [13,26,27]. Location-specific synthesis reinforces the general principle that both benefit and risk track the operative target rather than the approach as a whole [13,26].

Pooled safety data are reassuring within their limits. In the largest synthesis, postoperative complications were uncommon: CSF leak in 1% (among the 174 patients in whom it was assessed), wound infection in 3%, ptosis in 4%, diplopia in 6%, and medial gaze palsy in 9%, with visual improvement in 47% [14]. CSF leak, the outcome of greatest reconstructive concern, is examined separately in Section 8; a dedicated meta-analysis places it at 2.5% for the single corridor [12]. The minimally invasive design also delivers the practical benefits routinely emphasized in the reviews: avoidance of craniotomy and temporalis mobilization, minimal brain retraction, a favorable cosmetic result, short hospital stay, and rapid recovery, though these are reported qualitatively far more often than they are quantified [5,12].

The principal weakness of the outcome literature is temporal and definitional rather than a matter of any single adverse rate. Follow-up is short, with a median of 18 months in the comparative SOM data and a mean of 27.6 ± 15.1 months in the largest safety synthesis, well within the window in which meningioma recurrence and progression typically declare themselves [11,14]. Outcome definitions vary between series, nomenclature is inconsistent (Section 3), and residual disease is graded globally rather than by compartment (Section 6), so even the pooled estimates that exist are built on heterogeneous foundations. The honest summary is that ETOA has accumulated enough evidence to establish feasibility, short-term safety, and a favorable functional and cosmetic profile in selected patients, but not enough to define its indications precisely or to validate its long-term oncological performance. That gap is what motivates the limitations and future directions that follow.

## 8. Limitations

The limitations of ETOA fall into four groups—technical, reconstructive, ophthalmic, and evidentiary—several of which follow directly from the anatomy set out in Section 4. Technically, the corridor is narrow and, with a single external portal, affords far less instrument freedom than the endonasal route: endoscope, retractor, and instruments compete for one aperture, restricting bimanual dissection and, critically, the management of significant bleeding [7,8]. Maneuverability improves with orbitotomy enlargement or a biportal technique, but neither eliminates the constraint [48,49,50]. Compounding this is a steep learning curve: results remain concentrated in a few pioneering centers, higher-difficulty targets are explicitly reserved for high-volume groups, and the graded difficulty scales themselves testify to how much of the corridor’s safe use depends on accumulated expertise [14,21]. Much of the published outcome data is, in effect, pioneer-dependent, and may not generalize to the broader surgical community without deliberate training.

Reconstruction is the corridor’s central technical weakness. Because the vascularized nasoseptal flap central to endonasal repair is unavailable, closure depends on multilayer avascular grafting, and the resulting CSF leak risk is the complication that most shapes case selection and technique [5,8]. The magnitude of that risk is best understood through its classification: complications have been grouped into those related to lesion exposure, to lesion removal, and to osteo-dural reconstruction, the last of which is where leak arises [12]. For the single corridor, the problem is largely controlled, with pooled leak rates of 1–2.5% [12,14] and individual small series ranging from no leaks to isolated leaks managed by lumbar drainage [22,53], and in SOM the transorbital leak rate does not differ from transcranial surgery [2]. The unresolved case is the combined transorbital–endonasal configuration, whose pooled SOM leak rate of 20.3% is the highest of any configuration; the estimate is imprecise and not statistically significant, but it identifies multiportal reconstruction as the frontier the field has yet to close [2].

Ophthalmic and orbital morbidity is intrinsic to operating through the orbit. Diplopia, ptosis, extraocular muscle injury, gaze palsy, enophthalmos, and rarely visual loss are the characteristic risks, and they define the complication profile that distinguishes transorbital from transcranial series [14,26]. The reassuring qualifications are that most oculomotor deficits are transient and attributed to traction or edema rather than transection, and that both risk and severity concentrate in the extended cavernous and superior orbital fissure dissections rather than the standard corridor [14,26]. Dedicated ophthalmological assessment nonetheless captures deficits that pooled neurosurgical reporting can miss, and its wider adoption is necessary before the ocular safety of the approach can be regarded as fully characterized [13,27]. Intraoperative neuromonitoring of the ocular motor and trigeminal nerves may mitigate these risks but is not yet standard [54,55].

The anatomical ceilings described in Section 4 are limitations in their own right. Globe displacement of roughly a centimeter caps the working channel; the corridor is paramedian and ipsilateral, with poor access to midline and contralateral disease; posterior fossa extension is selective and usually contingent on petrosectomy or a combined route; and the cavernous carotid is not identified early because it lies behind the ophthalmic nerve [7,8,20]. Lesion factors compound these boundaries: bulky, firm, highly vascular, or vessel-encasing tumors are poorly suited to a corridor that limits both instrument exchange and hemorrhage control [7,8]. These are constraints on what can be safely reached and removed, not merely on convenience, and they are the principal determinants of the residual the approach leaves behind.

Finally, the evidentiary limitations are as consequential as the technical ones. There are essentially no comparative or long-term data outside a small number of retrospective meta-analyses; indications have not been consensus-defined; outcome reporting and nomenclature are inconsistent; and, as Section 6 argued, residual disease is reported globally rather than by anatomical compartment, which leaves the central oncological question about the approach formally unanswerable on present data [8,11,14,25]. These are not reasons to withhold the corridor, but they are the reasons its indications remain provisional, and they set the agenda for the directions considered in Section 10.

## 9. Comparative Context

ETOA is most usefully understood in relation to the two families of approaches it borders (Table 2). Against endoscopic endonasal surgery, the relationship is complementary rather than competitive, and the dividing line is anatomical: the endonasal corridor commands the ventral midline and the region medial to the optic nerve and internal carotid artery, whereas the transorbital corridor commands the territory lateral to them, the spheno-orbital region, lateral middle fossa, lateral cavernous wall, and Meckel’s cave, which the endonasal route reaches only by working across the very structures it must protect [4,5,7]. The two meet at defined connection zones and, for lesions that straddle the optic–carotid axis, can be combined in a single multiportal operation [9,10]. Direct comparisons for shared indications, such as trigeminal schwannoma and middle fossa tumors, accordingly conclude not that one corridor is superior but that corridor choice should follow tumor extent [60,61].

Against transcranial and lateral microsurgical approaches, the pterional, orbitozygomatic, and mini-pterional routes, the trade-off is different in kind. These provide wider exposure and a higher probability of gross total resection, at the cost of soft-tissue dissection, temporalis mobilization, variable brain retraction, and a cosmetic penalty [1,2,3]. ETOA inverts that balance, trading maximal resection for a shorter, retraction-free, cosmetically favorable route, and in SOM its lower GTR rate coexists with higher pooled proptosis relief and an overall complication rate that did not differ significantly from that of transcranial surgery [2,11]. These comparisons, however, derive from small, retrospective, non-randomized transorbital cohorts that differ systematically from transcranial cohorts in tumor extension, anatomical complexity, and selection, without adjustment for case mix; they indicate that the transorbital trade-off is reasonable in selected patients, not that ETOA is superior to transcranial surgery [2,11]. A single-center comparison with the extended mini-pterional approach frames the choice in the same terms [57]. As Section 6 argued, this trade-off is coherent only once the operative objective is fixed in advance: where the goal is decompression and functional preservation, the transorbital balance is attractive; where it is radical cytoreduction of bulky multicompartmental disease, a transcranial route remains preferable.

The correct framing, then, is neither ETOA as a replacement for open surgery nor as a rival to the endonasal route, but as one instrument in a multiportal armamentarium whose corridors are selected, and sometimes combined, according to where a lesion sits relative to the optic nerve and carotid, what the operative objective is, and what the tumor’s bulk and vascularity permit [8,67]. Its value lies in occupying an anatomical niche the other corridors serve poorly, not in displacing them.

## 10. Future Perspectives

The technological trajectory of ETOA is toward better visualization and finer control within a corridor whose defining problem is confined working space. High-definition 3D and 4K endoscopy, and exoscopic systems that decouple the optical axis from the surgeon’s line of sight, are natural fits for a deep, narrow channel, and robotic assistance has been proposed as a means of achieving stable, tremor-free instrument control where hand-and-endoscope crowding is most limiting; these remain aspirational for the transorbital corridor specifically and are advanced here as directions rather than established gains [8,67]. The nearer-term ergonomic advances are procedural rather than robotic; the biportal technique and calibrated orbitotomy enlargement already quantified as means of restoring surgical freedom [48,49,50].

Image guidance is likely to deepen. Navigation registered to preoperative CT, volumetric simulation of transorbital and endonasal views before multiportal surgery, and intraoperative CT to quantify residual between stages are already in use [6,7], and augmented-reality overlay of these data onto the operative field is a logical extension for a corridor in which key structures, most notably the cavernous carotid behind the ophthalmic nerve, are not seen early [7]. Custom angled instrumentation and endoscopes designed for the transorbital trajectory would address the same constraint from the mechanical side.

Indications will continue to expand, but the appropriate pace differs by domain. Vascular applications, carotid-cavernous fistula and, anatomically, exposure of the proximal middle cerebral artery, should advance cautiously, because the single portal that constrains hemorrhage control is most dangerous precisely where vascular work is performed [7,47,66]. Functional and temporal-pole applications rest largely on anatomical and early clinical evidence and warrant the same restraint [8,39,40]. Pediatric transorbital surgery has been reported and is promising, but remains, like the other higher-difficulty targets, the province of high-volume centers pending broader experience [21,41].

Two non-technological priorities may matter more than any device. The first is training and standardization: simulation and cadaveric curricula are the most direct means of flattening a learning curve that currently confines results to pioneers, and the graded-difficulty and target-zone frameworks provide a ready scaffold for structured, stepwise instruction [21,43,44]. The second is nomenclature: the interchangeable use of TONES, ETOA, SETOA, and their variants (Section 3) obstructs comparison across series, and the modular and target-based classifications already proposed offer a basis for the standardized vocabulary the field needs [5,8,25].

Above all, the evidence agenda is the field’s own stated priority. Prospective, comparative, multicenter studies and registries are required to move beyond the retrospective single-arm series that dominate the literature and to define indications with the precision that pooled feasibility data cannot provide [11,12,14]. Two reporting reforms would make that evidence far more useful and are achievable now: recording residual disease by anatomical compartment rather than as a global grade, using the volumetric and intraoperative-imaging methods already demonstrated [7,26], and documenting WHO grade and adjuvant treatment consistently, so that residual can be risk-stratified and multimodality outcomes interpreted [2]. Only with these in place can the central question the corridor raises, whether its larger residual carries an oncological cost, be answered.

To make that agenda concrete, Table 4 proposes a minimum reporting dataset for transorbital series. Its core is compartment-resolved grading of residual tumor, which replaces the single global label (gross total, near-total, or subtotal resection) that obscures where residual disease remains and conflates deliberately unresected tumor with incomplete resection. Residual tumor is recorded separately for each anatomical compartment (hyperostotic bone, intraorbital soft tissue, intracranial/dural, superior orbital fissure and cavernous sinus, and optic canal), together with whether residual in that compartment was planned, and is quantified on postoperative baseline imaging obtained at a stated interval [7,26]. Under this scheme, a case graded globally as “subtotal” may prove to represent complete decompression of the symptomatic compartments with intentional residual in the cavernous sinus, reframing an apparent oncological shortfall as a deliberate, function-preserving strategy. Ophthalmological outcomes, currently reported heterogeneously and often without dedicated ophthalmological examination [13,27], are standardized as best-corrected visual acuity, visual fields, exophthalmometry, ocular motility and diplopia, eyelid position, and pupillary function, each measured preoperatively and at fixed postoperative time points. Long-term tumor control requires WHO grade, adjuvant therapy, and radiographic progression defined by standardized criteria, such as those proposed by the Response Assessment in Neuro-Oncology (RANO) working group for meningioma, reported by compartment and with follow-up extending to at least five years [2,19,28]. None of these elements requires new technology, and each has already been applied individually in transorbital or meningioma series [7,26,27]; their joint adoption would allow future syntheses to compare approaches by compartment rather than by global grade.

## 11. Conclusions

The endoscopic transorbital approach has matured, in little more than a decade, from anatomical curiosity into an established corridor to the anterolateral skull base, occupying a niche, territory lateral to the optic nerve and carotid, reached without craniotomy, temporalis mobilization, or meaningful brain retraction, that neither the endonasal nor the transcranial route serves well. Its flagship indication, the spheno-orbital meningioma, is best approached not as a target for radical excision but as one for planned, decompression-oriented resection in anatomically selected patients, with adjuvant therapy decided before the operative approach is chosen. Judged against that objective, its short-term outcomes—favorable proptosis relief, acceptable visual and cranial-nerve results, low complication rates, and short recovery—are consonant with intent, even where its gross total resection rates trail those of open surgery. Whether its oncological results match those of open surgery remains unknown, and will remain so until case-mix-adjusted comparisons with long-term, compartment-specific follow-up are available.

The corridor’s future, however, depends less on extending its anatomical reach than on consolidating what it already does. Reconstruction, secure for the single corridor but unresolved for multiportal surgery, must be made reliable; the learning curve that confines results to a handful of pioneering centers must be flattened through simulation and standardized training; nomenclature and outcome sets must be harmonized; and the retrospective, short-follow-up, single-arm evidence base must give way to prospective, comparative, multicenter data that report residual by compartment and record grade and adjuvant treatment. These are the tasks that separate a promising corridor from a fully validated one. ETOA has convincingly demonstrated what it can reach; the work that remains is to prove, rigorously and comparably, how well it should be used.

## Figures and Tables

**Table 1:** Reported outcomes and complications of the ETOA across major syntheses, with transcranial comparators.

Synthesis (Year); Design	Patients, *n* (Studies, *n*)	Approach	GTR, %	Proptosis Improvement, %	Visual Improvement, %	CSF Leak, %	Overall Complications, %	Median Follow-Up, mo
Arend et al. (2026) [14]; SR and meta-analysis (2 prospective, 9 retrospective; 2019–2025)	269 (11)	ETOA	NR	NR	47	1	NR ^a^	NR
Corvino et al. (2025) [12]; SR and meta-analysis (CSF-leak endpoint)	240 (25)	ETOA	NR	NR	NR	2.5	NR	NR
Lam et al. (2025) [13]; SR (location-stratified)	382 (28)	ETOA	— ^b^	— ^b^	— ^b^	— ^b^	— ^b^	NR
Porto et al. (2026) [11], ETOA arm; SR and meta-analysis ^c^	155	ETOA	21.1	96	31	NR	18	18
Porto et al. (2026) [11], transcranial arm; SR and meta-analysis ^c^	1864	Transcranial	48.0	72	33	NR	26	52
Agosti et al. (2023); SR [2] and meta-analysis (SOM, all approaches)	1903 (59)	Mixed ^d^	57 ^e^	79.3	57.5	NR	NR	NR

Reported outcomes and complications of the ETOA across major syntheses, with transcranial comparators. Pooled proportions are shown as reported by each source; cells are marked NR where the outcome was not reported (or fell outside a focused synthesis’s scope). All included ETOA syntheses pool single-arm, predominantly retrospective series with short follow-up. Abbreviations: CSF, cerebrospinal fluid; ETOA, endoscopic transorbital approach; GTR, gross total resection; MTA, microsurgical transcranial approach; NR, not reported; SOM, spheno-orbital meningioma; SR, systematic review. ^a^ Arend et al. reported component complication rates rather than a single composite: wound infection 3%, ptosis 4%, diplopia 6%, medial gaze palsy 9%, transient facial numbness 16%, visual worsening 1%, CSF leak 1%, mortality 0%. ^b^ Lam et al. reported outcomes separately by anatomical group—orbital (*n* = 113), cavernous sinus (*n* = 58), extradural (*n* = 18), and intradural (*n* = 150)—rather than as pooled rates. ^c^ Porto et al. pooled 53 studies (2016 patients; 155 ETOA, 1864 transcranial) using random-effects generalized linear mixed models. ^d^ Agosti et al. pooled all approaches (MTA-dominant); GTR varied by approach from 23.5% (ETOA) to 59.8% (MTA). ^e^ Overall pooled GTR 57% (875/1542) across all approaches. CSF leak was assessed in 8 of the 11 included studies (174 of 269 patients); the meningioma-only subgroup rate was 6% (95% CI 2–14%; 4 studies, 92 patients). Between-approach comparisons (Porto et al.; Agosti et al.) are unadjusted for tumor class, extension, and baseline visual and proptotic status, and are subject to selection bias.

**Table 2:** Comparative profile of the transorbital, endonasal, and transcranial approaches.

Attribute	Endoscopic Transorbital (ETOA)	Endoscopic Endonasal (EEA)	Transcranial Microsurgical (MTA)
Trajectory/corridor	Lateral-to-medial through an orbital wall; no craniotomy	Ventral, midline transnasal–transsphenoidal; medial-to-lateral	Craniotomy (pterional, orbitozygomatic, mini-pterional); supero-lateral
Optimal targets	Spheno-orbital region, lateral sphenoid wing, lateral cavernous wall, middle fossa, Meckel’s cave, optic canal (lateral to optic nerve/ICA)	Ventral midline and paramedian disease—planum, tuberculum, medial cavernous sinus, medial optic canal (medial to optic nerve/ICA)	All compartments, including bulky intradural/intracranial disease and extensive dural involvement
GTR likelihood in SOM	Lower (pooled ≈ 21%); typically planned subtotal/decompressive	Intermediate; strongly lesion-dependent	Higher (pooled ≈ 48–60%)
Characteristic complications	Orbital/cranial-nerve—diplopia, ptosis, transient V2 hypoesthesia; low CSF-leak rate (≈1–2.5%)	CSF leak (comparatively higher), endocrine and sinonasal morbidity	Retraction injury, ophthalmoplegia, temporalis atrophy; broadest complication spectrum
Cosmesis	Favorable—eyelid/transorbital incision, no temporalis disinsertion	Favorable—no external incision	Least favorable—scalp incision, temporalis atrophy, potential contour defect
Reconstruction demand	Evolving; watertight closure remains challenging—principal current limitation	Established—nasoseptal flap, multilayer repair	Established—vascularized flaps, standard duraplasty
Learning curve	Steep; concentrated in a few high-volume centers	Steep but widely disseminated	Familiar to most neurosurgeons
Evidence maturity	Immature—single-arm retrospective series, short follow-up (median ≈ 18 mo)	Moderate—larger series, some comparative data	Mature—decades of data, longer follow-up (median ≈ 52 mo)

Comparative profile of the endoscopic transorbital, endoscopic endonasal, and transcranial approaches to the anterior and middle skull base. Entries are qualitative and reflect the consensus of the cited comparative and anatomical literature rather than a formal head-to-head analysis; pooled GTR ranges are drawn from the meta-analytic sources in Table 1. Abbreviations: EEA, endoscopic endonasal approach; ETOA, endoscopic transorbital approach; GTR, gross total resection; ICA, internal carotid artery; MTA, microsurgical transcranial approach; SOM, spheno-orbital meningioma.

**Table 3:** Practical indications and relative contraindications for the endoscopic transorbital approach compared with transcranial surgery.

Consideration	Favors ETOA	Favors a Transcranial (or Combined) Approach
Location relative to optic nerve and ICA	Lateral to the optic nerve and ICA: spheno-orbital region, lateral sphenoid wing, lateral orbit, middle fossa floor, lateral cavernous wall, Meckel’s cave [5,8]	Medial or inferomedial disease (endonasal preferred) or lesions crossing the optic–carotid axis (combined corridor); midline or contralateral extension; extensive anterior fossa or convexity component [5,8,9,10]
SOM anatomical class and morphology	Class I (lateral/superolateral) tumors; hyperostosis-dominant or en-plaque tumors with limited intradural component [2,16,17]	Diffuse (class IV) tumors and bulky intradural disease beyond the reach of the corridor [2,17]
Operative objective	Planned decompression of the orbit and optic canal, relief of proptosis and visual compromise, tissue diagnosis, functional preservation [2,11,18]	Maximal cytoreduction as the primary goal, e.g., suspected or known WHO grade 2–3 tumors, for which maximal safe resection is recommended [2,11,19]
Tumor characteristics	Small-to-moderate soft-tissue volume; bone-dominant disease; limited vascularity [7,8]	Bulky, firm, or highly vascular tumors; encasement of the ICA or its branches, or anticipated need for proximal vascular control [7,8]
Posterior and deep extension	Selected petrous apex or petroclival extension, typically with anterior petrosectomy and in high-volume centers [20,21]	Substantial posterior fossa extension beyond the trigeminal porus [8,20]
Prior treatment	Recurrence after prior transcranial surgery, offering an unscarred corridor (feasibility data only) [22]	Dense fibrosis after prior surgery and repeated radiosurgery, which may preclude interdural dissection [7]
Ocular status	Orbit tolerant of limited (≈1 cm) retraction; ophthalmological assessment available [23,24]	Ocular risk factors for retraction injury (e.g., high myopia, thin sclera, prior refractive or glaucoma surgery) warrant ophthalmological evaluation and may favor an alternative route [24]
Reconstruction	Defects amenable to multilayer closure through the orbital wall [12,14]	Anticipated large dural defect communicating with the paranasal sinuses or cribriform region, where vascularized reconstruction is required [5,8]
Institutional experience	Team with transorbital experience and ophthalmology support, particularly for higher difficulty levels [14,21]	Limited institutional transorbital experience with a target of high difficulty level [21]

Practical indications and relative contraindications for the endoscopic transorbital approach compared with transcranial surgery. Entries are relative rather than absolute and summarize the anatomical, technical, and observational literature cited; they are not derived from randomized or case-mix-adjusted comparisons. Abbreviations: ETOA, endoscopic transorbital approach; ICA, internal carotid artery; SOM, spheno-orbital meningioma; WHO, World Health Organization.

**Table 4:** Proposed minimum reporting dataset for endoscopic transorbital series.

Domain	Element	Suggested Definition and Timing
Patient and tumor	Anatomy and extent	SOM class (I–IV) [2]; compartments involved preoperatively; tumor and hyperostosis volume on preoperative MRI/CT
	Histology	WHO grade [19]
Operative	Corridor and extension	Quadrant of entry; standard vs. extended (proximal/distal) approach [25]; level of difficulty [21]; target zone [26]; single vs. combined/multiportal corridor
	Surgical intent	Planned decompression vs. attempted complete removal, specified for each compartment before surgery
	Reconstruction	Technique and materials; CSF leak and its management
Residual tumor by compartment	Hyperostotic bone; intraorbital soft tissue; intracranial/dural; superior orbital fissure/cavernous sinus; optic canal	Present/absent and volume for each compartment; planned vs. unplanned; postoperative baseline MRI (with CT for bone) at a stated interval [7,26]
Ophthalmological outcomes	Visual acuity and visual field	Best-corrected visual acuity (logMAR); standard automated perimetry
	Proptosis	Exophthalmometry (mm), relative to the contralateral eye
	Motility, eyelid, pupil	Ductions and diplopia; ptosis; relative afferent pupillary defect
	Timing and examiner	Preoperatively, early postoperatively, at 3–6 months, and at ≥12 months; ophthalmologist-performed where possible [13,27]
Complications	Cranial nerve and orbital	Deficits by nerve, graded as transient (<6 months) or permanent; enophthalmos; temporal hollowing; wound complications
Long-term tumor control	Progression	Radiographic progression by standardized criteria (e.g., RANO meningioma), reported by compartment [28]
	Adjuvant therapy	Modality, timing (planned vs. at progression), and dose [19]
	Follow-up	Median and range; progression-free survival at 2, 5, and 10 years; time to retreatment; minimum of 5 years for tumor-control endpoints

Proposed minimum reporting dataset for endoscopic transorbital series. The dataset is proposed by the authors on the basis of the cited classifications and of the reporting gaps identified in this review; time points are suggestions intended to harmonize future series. Abbreviations: CSF, cerebrospinal fluid; logMAR, logarithm of the minimum angle of resolution; RANO, Response Assessment in Neuro-Oncology; SOM, spheno-orbital meningioma; WHO, World Health Organization.

## Data Availability

No new data were created or analyzed in this study. Data sharing is not applicable to this article.
